# Carboxypeptidase G and pterin deaminase metabolic pathways degrade folic acid in *Variovorax* sp. F1

**DOI:** 10.1186/s12866-022-02643-6

**Published:** 2022-09-27

**Authors:** Yungmi You, Yuki Doi, Norifumi Maeda, Shunsuke Masuo, Norio Takeshita, Naoki Takaya

**Affiliations:** Faculty of Life and Environmental Sciences, Microbiology Research Center for Sustainability, University of Tsukuba, 1-1-1 Tennodai, Tsukuba, Ibaraki 305-8572 Japan

**Keywords:** Vitamin B_9_, Pterin deaminase, Pteroic acid, Deaminofolic acid, *Variovorax*

## Abstract

**Background:**

Folic acid (FA) is a synthetic vitamin (B_9_) and the oxidized form of a metabolic cofactor that is essential for life. Although the biosynthetic mechanisms of FA are established, its environmental degradation mechanism has not been fully elucidated. The present study aimed to identify bacteria in soil that degrade FA and the mechanisms involved.

**Results:**

We isolated the soil bacterium *Variovorax* sp. F1 from sampled weed rhizospheres in a grassland and investigated its FA degradation mechanism. Cultured *Variovorax* sp. F1 rapidly degraded FA to pteroic acid (PA), indicating that FA hydrolysis to PA and glutamate. We cloned the carboxypeptidase G (CPG) gene and found widely distributed paralogs within the *Variovorax* genus. Recombinant CPG preferred FA and deaminofolic acid as substrates, indicating its involvement in FA degradation by *Variovorax*. Prolonged culture of *Variovorax* sp. F1 resulted in decreased rates of deaminofolic acid (DFA) and deaminopteroic acid (DPA) accumulation. This indicated that the deamination reaction also comprised a route of FA degradation. We also identified an F1 gene that was orthologous to the pterin deaminase gene (Arad3529) of *Agrobacterium radiobacter*. The encoded protein deaminated FA and PA to DFA and DPA, which was consistent with the deamination activity of FA and PA in bacterial cell-free extracts.

**Conclusion:**

We discovered that the two enzymes required for FA degradation pathways in isolates of *Variovorax* sp. F1 comprise CPG and pterin deaminase, and that DFA and PA are intermediates in the generation of DPA.

**Supplementary Information:**

The online version contains supplementary material available at 10.1186/s12866-022-02643-6.

## Introduction

Vitamins are essential coenzymes that regulate cellular metabolism. Humans and other animals cannot synthesize most essential vitamins so they must be ingested from foods, to avoid deficiencies that can cause various symptoms and pathological states [1, 2]. Thus, the physiological functions of vitamins have been established [3]. Autotrophic plants and microorganisms produce vitamins via known synthetic mechanisms. Chemically synthesized vitamins support an increasing demand for food supplements [4–6]. The amounts of vitamins in living organisms are homeostatic and maintained, but the molecular mechanisms of vitamin decomposition by ecological systems are not fully understood. Examples are recent findings of bacterial genes encoding proteins that degrade thiamine (vitamin B_1_) [7], riboflavin (vitamin B_2_) [8], nicotinamide (vitamin B_3_) [9], pyridoxine (vitamin B_6_) [10], and L-ascorbate (vitamin C) [11, 12].

Folic acid (FA, vitamin B_9_) is a synthetic N-pteroyl-p-aminobenzoylglutamate and precursor of tetrahydrofolic acid (THFA). Its related compounds are involved in cellular one-carbon metabolism [13, 14], and it is essential for human functions. A deficiency of THFA and its related compounds results in anemia, stunted growth, and neural tube defects in newborns. Folic acid is therefore a therapeutic agent and nutrient supplement that is particularly important for pregnant women [15, 16]. Folic acid is chemically synthesized on an industrial scale, whereas its microbial fermentation is under development [17]. The structure of FA comprises pterin, *p*-aminobenzoic acid (PABA), and glutamate moieties [18]. The de novo biosynthetic pathway of THFA starts from guanosine triphosphate (GTP), and mediates dihydropterin, which is conjugated with *p*-aminobenzoic acid and glutamate to generate the reduced forms of FA, dihydrofolic acid and tetrahydrofolic acids. Multiple conjugation reactions of glutamate generate polyglutamate derivatives. The extent of polyglutamylation varies among species and it regulates cofactor affinity for enzymes and subcellular compartmentation in plants [19].

Despite extensive investigation into biosynthesis of THFA and its related compounds, the mechanisms of their degradation in animals and the environment are not fully understood. Tetrahydrofolic acids and their products derived from plants and microorganisms like other natural compounds, are considered to be decomposed in the material cycles of soil. Tetrahydrofolic acids are quite easily oxidized to FA in fertile aerobic soils. Therefore, elucidating the taxonomic distribution and physical location of bacteria that degrade FA is important for understanding microbial community involvement in the FA material cycle. Furthermore, understanding the bacterial enzymes and genes responsible for FA degradation is important to determine the molecular mechanisms of THFA mineralization by soil ecosystems.

A Pseudomonad bacterium isolated in 1967 hydrolyzed FA to pteroic acid (PA) and glutamate [20], then a protein in the carboxypeptidase G family (CPG; EC 3.4.17.11) catalyzing this reaction was identified [20–23] and their encoding genes were cloned from several *Pseudomonas* species [24]. Pterin deaminase (PDA; EC 3.5.4.11) deaminates FA to deaminofolic acid (DFA) in cell-free extracts of *Alcaligenes metalcaligenes* [25], *A. faecalis* [26], *Flavobacterium polyglutamicum* [27], *Pseudomonas* sp. Fo8 [28], and *Bacillus megaterium* [29]. Genetic evidence of bacterial PDA for FA had remained obscure before homology modeling and identification of the Arad3529 protein from *Agrobacterium radiobacter* K84 that deaminates FA [30]. Some PDA deaminates PA to deaminopteroic acid (DPA) [25, 26, 28], but whether PDA participates in PA deamination in *A. radiobacter* K84 cells has remained obscure. *N*-(4-aminobenzoyl)-L-glutamic acid (ABG) is an oxidation product of THFA [31], and *Escherichia coli* BN101 produces its ABG hydrolase, AbgAB, that seems inert against FA [32]. Besides these findings, bacteria that utilize both a carboxypeptidase and a deaminase to degrade FA are limited to the tentatively classified *Pseudomonas* sp. Fo8 that was discovered in 1974 [28]. However, the culture properties of this bacterium, the genetic basis of the mechanism of FA degradation and its classification have remained unknown.

Here, we screened a series of soil samples for FA-degrading bacteria. We identified the novel FA-degrading bacterium *Variovorax* sp. F1, which consumed FA and accumulated PA in vitro, indicating that it utilizes glutamate liberated from FA. The accumulation of both DFA and DPA in cultures indicated that *Variovorax* sp. F1 deaminates FA and PA. We cloned the *Variovorax* sp. F1 genes for CPG and PDA, and recombinant CPG (rCPG) that hydrolyzed FA and deaminofolic acid (DFA). Recombinant PDA (rPDA) deaminated FA and PA to DFA and DPA, respectively, indicating two pathways of FA degradation in the soil bacterium *Variovorax* sp. F1.

## Results

### Isolation of soil bacteria that degrade FA

**Fig. 1:**
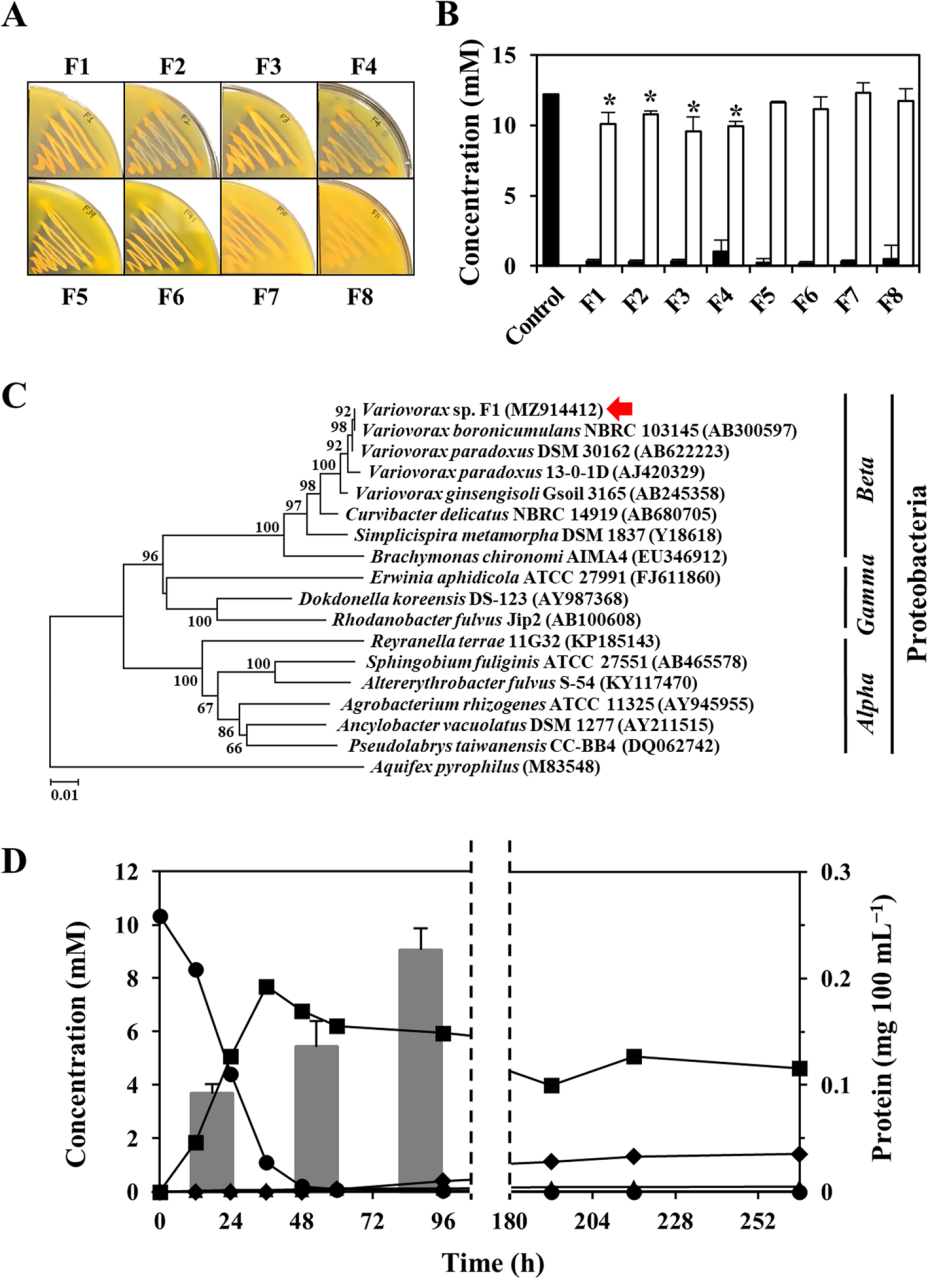
Isolation of FA degrading bacteria. **A** Growth of eight isolates on M9-FA agar plates at 28 °C for 48 h. **B** Degradation of FA to PA by isolates cultured in M9-FA medium at 28 °C for 48 h. Filled bars, FA; unfilled bars, PA. Error bars, standard errors of means (*n* = 3, *p* < 0.05). *P < 0.05 (control FA vs. accumulated PA). **C** Nucleotide sequences of selected strains were aligned, and phylogenetic trees were constructed by neighbor-joining method [33] using MEGA X software [34]. Numbers along branches indicate 1000 bootstrap replicates **D** Time-dependent decomposition of FA by typical culture of *Variovorax* sp. F1 in M9-FA medium at 28 °C for 48 h. ●, FA; ■, PA; ▲, DFA; ◆, DPA. Bars, cell growth estimated as total cellular proteins. Error bars, standard errors of means (*n* = 3). DFA, deaminofolic acid; DPA, deaminopteroic acid; FA, folic acid; PA, pteroic acid

Six environmental soil samples were cultured in M9 Minimal Medium containing FA as the sole carbon source (M9-FA medium) to enrich bacteria that can degrade FA. Orange FA disappeared and yellow insoluble pigments appeared on plates containing M9-FA agar inoculated with eight cultured samples (Fig. 1A). Cultures in liquid M9-FA medium also consumed FA to form yellow insoluble pigments, which were solubilized in 0.1 M NaOH, and confirmed as PA by high performance liquid chromatography (HPLC) equipped with an alkaline-tolerant column and a photodiode array detector. All isolates consumed most of the FA and some accumulated less PA than the amount of consumed FA (Fig. 1B). We further analyzed F1 among the eight strains. The nucleotide sequence of the 16S rRNA gene of the F1 strain was 99.9% identical to that of *Variovorax paradoxus* NBRC 15149^T^ and *V. boronicumulans* NBRC 103145^T^. Phylogenetic analyses indicated that the F1 strain is a β-proteobacterium related to the genus *Variovorax* (Fig. 1C) and was identified as *Variovorax* sp. F1. Partial sequencing of the 16 S rRNA gene revealed that the other seven isolates were related to the *Variovorax* and *Xenophilus* genera and belonged to the *Comamonadaceae* family (Table S1).

**Fig. 2:**
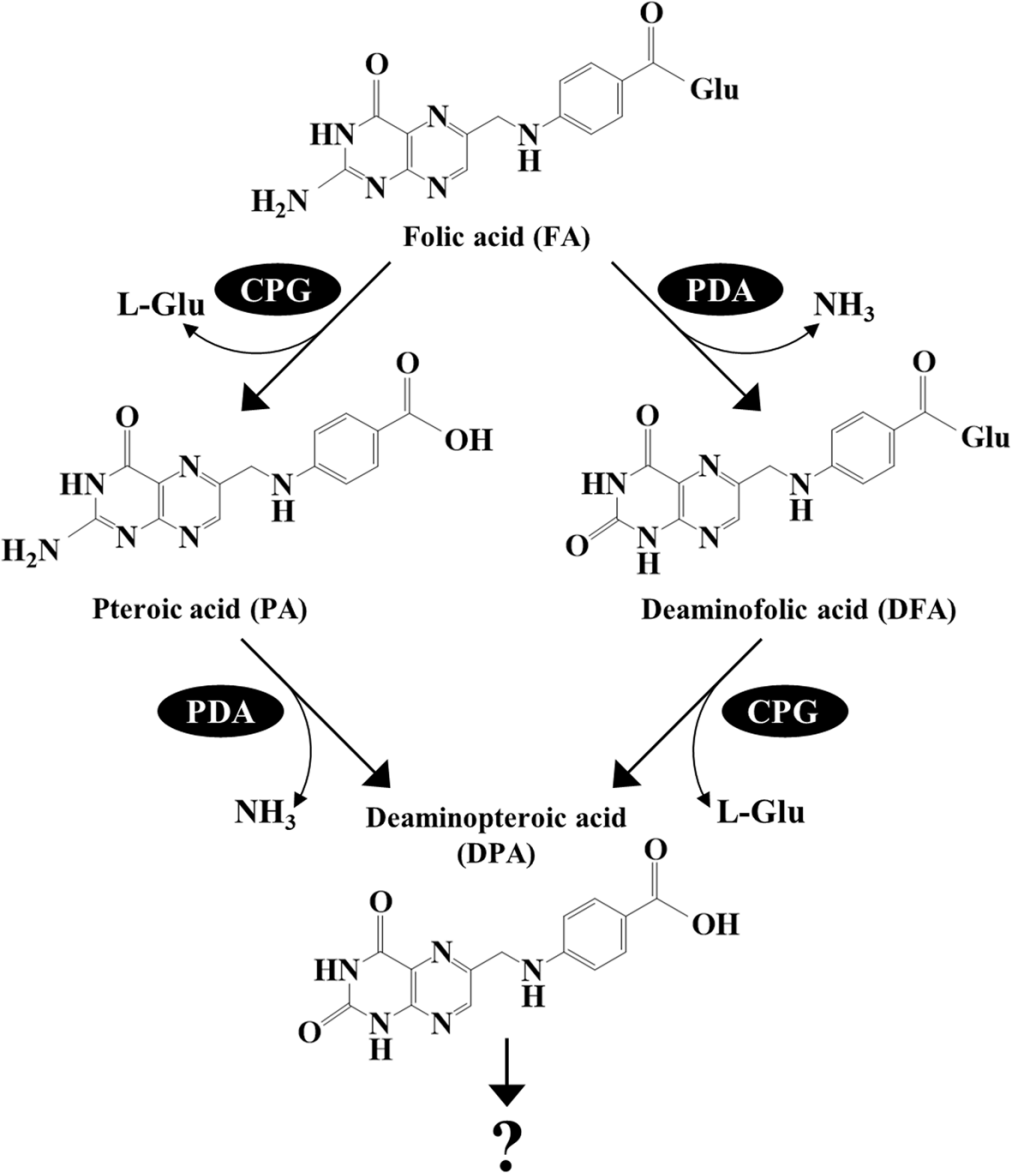
Proposed FA degradation pathway in *Variovorax* sp. F1. Schematic model of FA degradation by *Variovorax* sp. F1. ABG, *N*-(4-aminobenzoyl)-L-glutamic acid; CPG, carboxypeptidase G; DFA, deaminofolic acid; DPA, deaminopteroic acid; FA, folic acid; PA, pteroic acid; PABA, *p*-aminobenzoic acid; PDA, pterin deaminase

We investigated time-dependent changes in FA and its degradation products in cultured F1 (Fig. 1D). This strain consumed > 90% of the initial 10 mM FA within 36 h when cultured in liquid M9-FA medium. The accumulation of ~ 8 mM PA indicated that the strain almost stoichiometrically converted FA to PA. The growth of *Variovorax* sp. F1 was measured as total protein in cultures using the Bradford method because insoluble FA and PA interfere with conventional measurements of cell mass based on optical density. The results indicated concomitant cell growth and FA conversion to PA, which implied that *Variovorax* sp. F1 degrades FA to PA and glutamate and utilizes it as a carbon source for growth. This notion is supported by fact that the rates of F1 growth were similar in medium containing glutamate as carbon and nitrogen sources and FA as a carbon source (M9-FA medium). Analysis of prolonged cultures using HPLC produced additional compounds that eluted at the same retention times as DFA and DPA prepared and identified herein (see Materials and methods). Incubation for 36 h resulted in the relatively low accumulation of DFA and DPA (~ 70 and < 5 μM, respectively), whereas culture for 264 h increased these amounts to 180 μM and 1.4 mM, respectively. These results suggested that the F1 strain respectively hydrolyzed FA and DFA to PA and DPA, then deaminated FA and PA to DFA and DPA (Fig. 2).

### *Variovorax* sp. F1 CPG for FA utilization

**Fig. 3:**
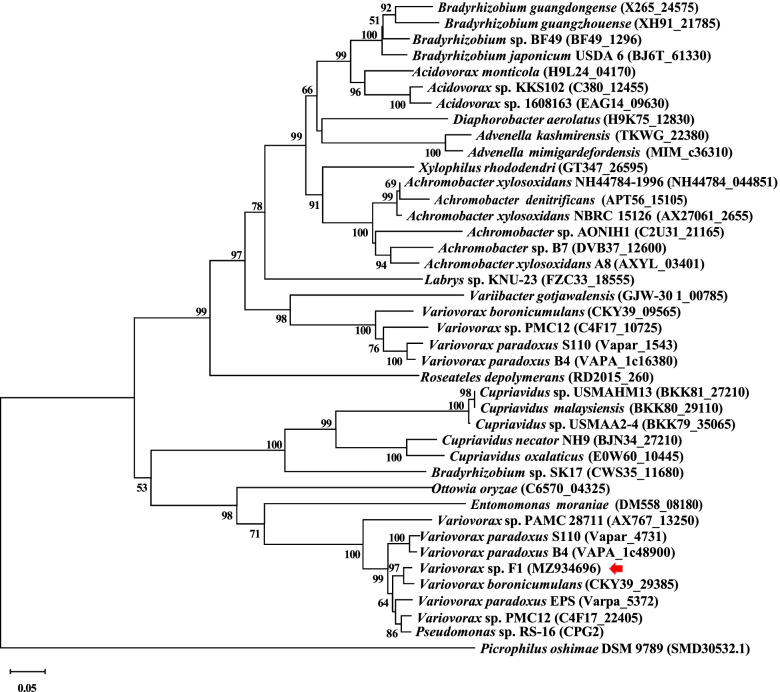
Phylogenetic relationship of CPG from *Variovorax* sp. F1. Amino acid sequences of predicted CPG from selected strains were aligned and phylogenetic trees were constructed by neighbor-joining [33] using MEGA X software [34]. Numbers along branches indicate 1000 bootstrap repeats. Gene IDs are shown after strain names in parenthesis

We considered that *Variovorax* sp. F1 would produce a counterpart of CPG2 in *Pseudomonas* sp. RS-16 [23] and other bacteria [22–32, 35, 36] to hydrolyze the C-terminal glutamate portion of FA. We therefore cloned the CPG2 paralog of the F1 strain. Database searches predicted CPG proteins from the *Variovorax* spp. SCN67–85, KB5, OV084, YR634, and root434, with amino acid sequence identities of 94.0–95.2%. We amplified the *Variovorax* sp. F1 CPG gene by PCR using custom-designed primer sets for conserved nucleotide sequences and total DNA of *Variovorax* sp. F1. The gene comprised an open-reading frame encoding 415 amino acids that was 93.5% identical to CPG2 of the most intensively studied *Pseudomonas* sp. RS-16 [37], and 94.7 and 97.6% identical respectively, to the CPGs predicted from *V. paradoxus* strain EPS (Varpa5372) and *V. boronicumulans* (CKY39_29385). The results of phylogenetic analyses showed that the CPG of the F1 strain was the most closely related to the predicted CPG of *V. boronicumulans* and *V. paradoxus* (Fig. 3). The phylogenetic tree contained more than one CPG from individual *Variovorax* strains in different clades. The amino acid sequence of the CPG of the F1 strain was 44.1–45.9% identical to those of CPGs with uncharacterized FA hydrolytic capacity located in different clades.

**Fig. 4:**
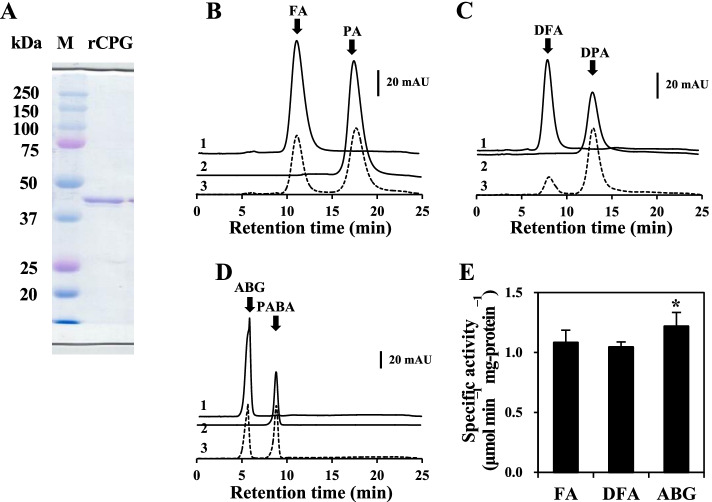
Enzymatic properties of rCPG derived from *Variovorax* sp. F1. **A** SDS-PAGE gel. Lanes: M, molecular weight marker; rCPG, Recombinant CPG (1.8 μg) from *Variovorax* sp. F1 strain. **B**–**D** HPLC analyses of reactions containing 5 μg mL^− 1^ rCPG and various substrates at 30 °C for 15 min. Substrates (5 mM) each are **B** FA; **C** DFA; **D** ABG. Traces: 1, substrates; 2, products; 3, reaction. **E** Specific activities of rCPG against FA, DFA, and ABG. Error bars indicate standard errors of means (*n* = 3). *P < 0.05. ABG, *N*-(4-aminobenzoyl)-L-glutamic acid; DFA, deaminofolic acid; DPA, deaminopteroic acid; FA, folic acid; PA, pteroic acid; rCPG, recombinant carboxypeptidase G

We generated a recombinant CPG (rCPG) of *Variovorax* sp. F1 with a 6 × histidine tag on the amino terminus using an *E. coli* expression system. The purified rCPG resolved on SDS-PAGE as a single band at 45 kDa (Fig. 4A). The reaction of rCPG and FA generated PA (Fig. 4B), indicating that rCPG hydrolyzes FA to PA and glutamate like the *Pseudomonas* CPG2. The initial velocity of the reaction against 5 mM FA was 52 ± 3 μmol min^− 1^ mg^− 1^ (Fig. 4B), and comparable to that of the known CPG (40–725 μmol min^− 1^ mg^− 1^) [21–23]. The reaction was inhibited by 1 mM EDTA, which agrees with the Zn^2+^-dependent reaction of *Pseudomonas* CPG2 [23].

### Substrate specificity of rCPG

The substrate specificity of rCPG against ABG derivatives was investigated considering that rCPG hydrolyzes FA at the amide bond within the ABG residue. The substrates FA and ABG are commercially available, and we prepared DFA in-house. Briefly, we deaminated commercial FA in a preparative scale using recombinant PDA from the F1 strain to obtain DFA and purified it to > 99%. The reaction of rCPG and DFA generated another compound on HPLC analysis (Fig. 4C). This compound eluted at the same retention time as DPA, which we prepared and identified from a parent mass ion peak and a mass ion fragment at *m*/*z* = 312.2 ([M–H]^−^) and 176.1, respectively, using LC-MS/MS (Fig. S1A; Materials and methods). These results indicated that rCPG hydrolases DFA to DPA.

Reactions of rCPG with FA, DFA and ABG (0.1 mM each) produced stoichiometric amounts of DPA and PABA (Fig. 4C, D) at rates of 1.0 ± 0.1 and 1.2 ± 0.1 μmol min^− 1^ mg^− 1^, respectively (Fig. 4E). These results indicated that FA, DFA and ABG are substrates of rCPG. The initial velocity of the rCPG reaction linearly increased as substrate concentrations increased to 30, 50, and 20 mM FA, DFA, and ABG, respectively (Fig. S2). Since the initial velocities of the enzyme did not reach saturation according to substrate concentrations, the Michaelis (*K*_m_) and kinetic (*k*_cat_) constants for these reactions could not be determined.

### Identification of PDA from *Variovorax* sp. F1

**Fig. 5:**
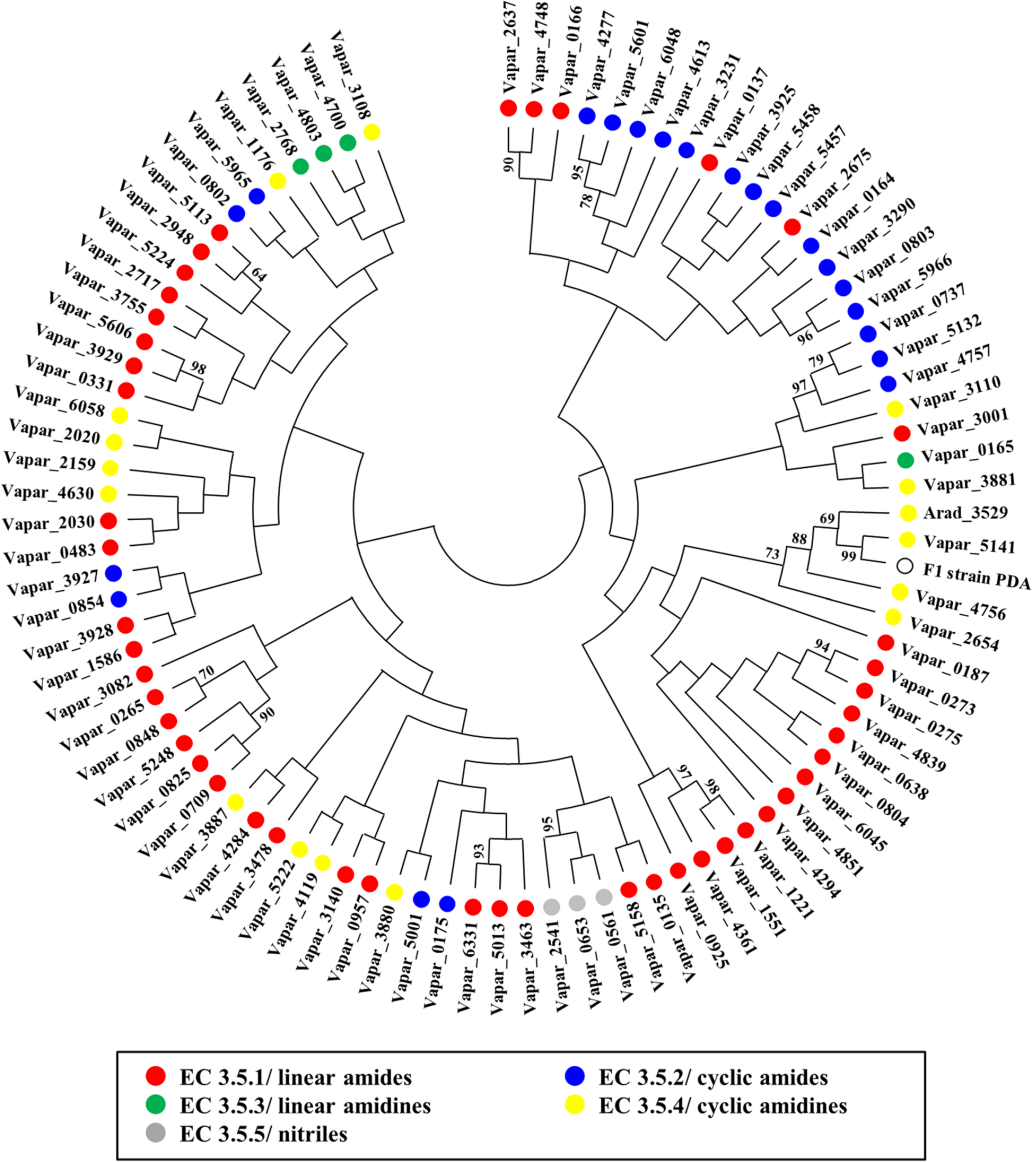
Phylogenetic relationships of deaminase from *Variovorax* sp. F1 PDA. Amino acid sequences of putative hydrolases that catalyze non-peptide carbon-nitrogen bond cleavage (EC 3.5) from *V. paradoxus* S110 were aligned, and phylogenetic trees were constructed by neighbor-joining [33] using MEGA X software [34]. Numbers along branches indicate 500 bootstrap repeats. Gene IDs are color-coded according to their enzyme families as: hydrolases acting on linear amides (red), cyclic amides (blue), linear amidines (green), cyclic amidines (yellow), and nitriles (gray)

The accumulation of DFA and DPA in culture medium of the F1 strain (Fig. 1D) indicated their production via FA and PA deamination. We searched for a *Variovorax* F1 protein orthologous to *A. radiobacter* K84 PDA (Arad3529) which was the only PDA identified to date as an enzyme that deaminates FA to DFA. Database searches identified a predicted deaminase (WP 068679287) in many *Variovorax* spp. that has 36.0% amino acid sequence identity with Arad3529. We aimed to construct a set of primers with a conserved nucleotide sequence among *V. paradoxus* orthologs to clone the corresponding gene in F1 strain. However, we found a conserved sequence for the primer corresponding to the 5′-, but not the 3′-end of the gene. We therefore designed a 3′-end primer with reference to conserved endoribonuclease genes located downstream of the predicted PDA genes in the *V. paradoxus* genome. The *Variovorax* sp. F1 PDA gene was amplified by PCR using the primers for the conserved nucleotide sequences and total DNA of *Variovorax* sp. F1. The DNA fragment contained a gene encoding 399 amino acid residues that were 76.7 and 37.0% identical, respectively, to the protein predicted from *V. paradoxus* S110 (Vapar5141) and Arad3529. These proteins were hydrolases that act on non-peptide carbon-nitrogen bonds (EC 3.5), which are diverse among bacteria. The *Variovorax paradoxus* S110 genome encodes 89 such hydrolases. Our phylogenetic analyses classified many of these enzymes based on the molecular structures of the substrates that they hydrolyze (Fig. 5). *Variovorax* sp. F1 PDA and Arad3529 were located in a branch of putative hydrolases that act on cyclic amidines (EC 3.5.2).

**Fig. 6:**
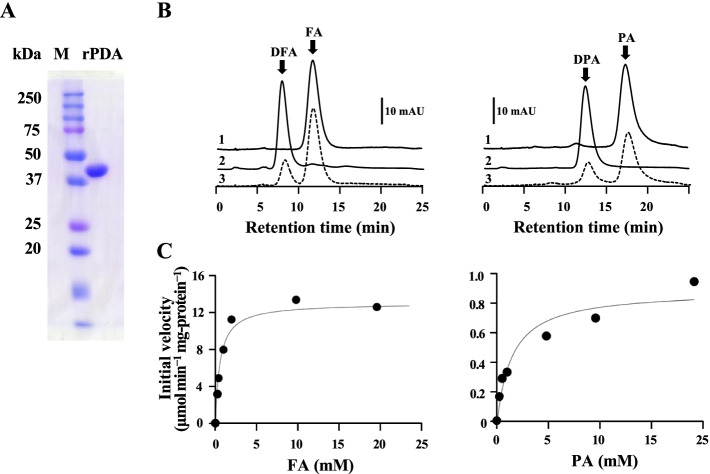
Enzymatic properties of rPDA from *Variovorax* sp. F1. **A** SDS-PAGE gel. Lanes: M, molecular weight marker; rPDA, Recombinant PDA (2 μg) from *Variovorax* sp. F1 strain. **B** HPLC analyses of PDA reactions containing 0.01 μg mL^− 1^ rPDA and 1 mM substrate (*left*, FA; *right*, PA) at 30 °C for 2 min. Traces: 1, substrates; 2, products; 3, reaction. **C** Initial velocity of rPDA reaction determined to calculate *K*_m_ and *k*_cat_ values of rPDA for FA (*left*) and PA (*right*) as substrates. Data were fitted to Michaelis-Menten equation

**Fig. 7:**
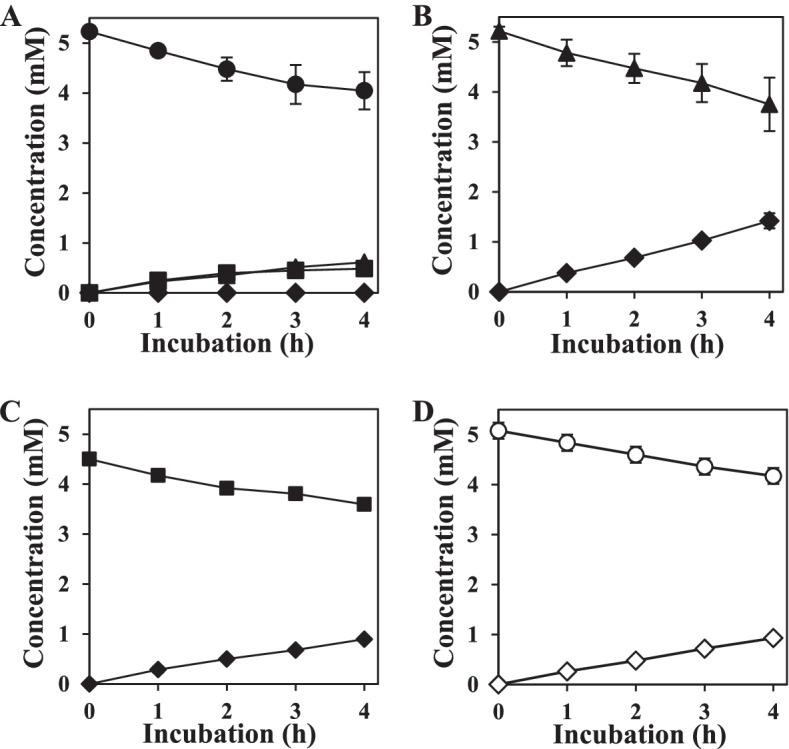
Reactions of cell-free extract of *Variovorax* sp. F1 with various substrates. Folic acid (**A**) degradation of DFA (**B**), PA (**C**), and ABG (**D**) in cell-free extracts prepared from F1 strain cultured in M9-FA medium. Reactions proceeded in 50 mM Tris-HCl (pH 7.5) containing 0.2 mM ZnSO_4_, cell-free extract (5 μg mL^− 1^ protein) and substrates at 30 °C for 4 h. No DPA was degraded. ●, FA; ■, PA; ▲, DFA; ◆, DPA; ○, ABG; ◇, PABA. Error bars, standard errors of means (*n* = 3)

We produced recombinant PDA (rPDA) from *Variovorax* sp. F1 using an *E. coli* expression system. Purified rPDA resolved as a single 45 kDa band on SDS-PAGE (Fig. 6A). The reaction between rPDA and FA generated a novel compound that was separated by HPLC (Fig. 6B, *left*). The LC-MS findings showed a parent mass ion peak at *m*/*z* = 441.2 ([M–H]^−^), which corresponded to the molecular mass (M*r* 442) of DFA (Fig. S1B). The mass ion fragments at *m*/*z* = 312.2 and 176.0 generated by this compound were consistent with the structure of deaminated pterin (Fig. S1B). The reaction of rPDA and PA generated another compound (Fig. 7C), which eluted at the same retention time as DPA (Fig. S1A). These results showed that this novel enzyme deaminated FA and PA and was *Variovorax* sp. F1 PDA.

### Steady-state kinetics of rPDA

The initial velocity of the PDA for deaminating FA and PA (1 mM each) was respectively 11.7 ± 1.2 and 0.31 ± 0.05 μmol min^− 1^ mg^− 1^. The reaction kinetics fit the Michaelis-Menten equation, with *K*_m_ and *k*_cat_ values of 0.28 ± 0.06 mM and 10.1 ± 0.4 *s*^− 1^ for FA, and 1.5 ± 0.6 mM and 0.62 ± 0.06 *s*^− 1^ for PA (Fig. 6C). The *K*_m_ values were comparable to the concentrations of FA and PA in the cultures. These results indicated that *Variovorax* sp. F1 PDA uses both FA and PA as substrates but preferentially deaminates FA. This finding supports the phylogenetically close relationship of F1 PDA to the predicted cytosine and creatine amidases (Vapar3881, Vapar4756, Vapar2654), the latter of which shares a guanidine moiety with pterins that are deaminated by PDAs.

### Cell-free activity reveals potential FA-degradation mechanism

The discovery of CPG and PDA genes in the F1 strain and accumulation of DFA and DPA in the culture suggested that the CPG-dependent cleavage of glutamate residues and deamination of the pterin moiety constitute an FA-degradation pathway. Therefore, we validated these activities in cell-free extracts of the cultured F1 strain that degraded FA. Reactions between cell-free extracts and FA resulted in decreased FA with a specific activity of 49 ± 2 nmol min^− 1^ mg^− 1^ (Fig. 7A). The reaction products were identified as essentially equal amounts of PA and DFA, indicating that the cells had CPG and PDA activities. Reaction with the cell-free extract resulted in the stoichiometric conversion of DFA to DPA (Fig. 7B) The reaction rate was faster than that for FA hydrolysis to PA (59 ± 6 vs. 20 ± 1 nmol min^− 1^ mg^− 1^; Fig. 7A), which agreed with our findings that bacterial rCPG hydrolyzed FA and DFA (Fig. 4D). We also found PA deamination activity in cell-free extracts with a specific activity of 37 ± 2 nmol min^− 1^ mg^− 1^ (Fig. 7C). This reaction produced DPA, which was the final product of the long-term culture (Fig. 1C). The cell-free extracts decomposed minimal amounts of DPA under these reaction conditions. Reactions between the cell-free extract and ABG, which is a product of THFA oxidation [31], generated PABA at a rate of 39 ± 1 nmol min^− 1^ mg ^− 1^ (Fig. 7D). These results indicated that the F1 strain degrades FA to DPA, and that PA and DFA are intermediates in the mechanism of bacterial FA degradation (Fig. 2).

## Discussion

Ecological systems maintain the homeostasis of almost all types of biological molecules including those that are bulk-produced to generate physiologically active compounds. Among them, FA is a synthetic vitamin and popular food supplement that natural microorganisms can decompose. This study found that *Variovorax* sp. F1 in soil degrades FA via two distinct CPG and PDA pathways that mediate PA and DFA to produce DPA and supply this bacterium with glutamate as carbon/energy sources for growth. These findings are consistent with bacterial CPG hydrolysis, FA deamination, and FA degradation to DPA that have not been explored for decades [28]. Conventional HPLC using acidic water and solvents is inappropriate for analyzing extremely soluble FA and related compounds. An alkaline-tolerant HPLC system enabled analysis of alkali-soluble compounds and their high-throughput quantitation. Consequently, we could identify the genus, enzymes, and culture properties associated with bacterial FA degradation.

Bacterial FA degradation was long considered to involve CPG and deaminase, but the reconstitution of reactions by two enzymes originating from a single *Variovorax* species is novel. The CPG of *Variovorax* sp. F1 was phylogenetically similar to that of *Pseudomonas* sp. RS-16 (CPG2) and explains why FA breakdown generated PA as a major product at a relatively rapid rate (~ 48 h; Fig. 1D). We found that DFA and DPA were also degradation products of FA (Fig. 2), indicating that the bacterium deaminated FA before CPG decomposed DFA to DPA. Time-dependent changes in the accumulation of less DFA and DPA (deaminase products) than PA (CPG product) in the culture broth revealed a slower deamination rate than that catalyzed by CPG (Fig. 1D). We reconstituted the bacterial PDA deamination reactions of FA and PA to DFA and DPA, respectively (Figs. 6 and 7). As far as we can ascertain, this is the first PDA for which the gene was cloned from FA-degrading bacteria, and the second example of a PDA with amino acid sequences that are similar to those of *A. radiobacter* PDA (Arad3529). The F1 PDA was located in the same branch as other predicted *Variovorax* proteins and Arad3529 (Fig. 5). Thus, our findings will enable the future discovery of other novel PDAs (EC 3.5.4.11) from proteins that are predicted deaminases for cyclic amidines (EC 3.5.4).

Cultured *Variovorax* sp. F1 consumed more FA than the degraded products (PA, DFA, and DPA) identified herein (Fig. 1C), indicating that this bacterium further catabolized these compounds. Lumazine-6-carboxylic acid and/or ABG are also candidate FA degradation intermediates that might be similarly generated in some bacteria [28]. The oxidative cleavage of DFA, DPA, and FA followed by deamination might generate lumazine-6-carboxylic acid. The rapid catalytic activity of rCPG against ABG (Fig. 5D) indicated the bacterial degradation of ABG to PABA and glutamate. Neither ABG nor lumazine-6-carboxylic acid were detectable in the cultured F1 strain, hence the mechanism of their production in this strain remains elusive.

The degradative mechanism of FA in ubiquitous *Variovorax* soil bacteria is likely to participate in the natural homeostasis of FA, THFA, and related compounds. Our extensive screen of sampled weed rhizospheres resulted mostly in *Variovorax* species (Table S1) and proteins with high similarity to CPG encoded by the *Variovorax* genome (Fig. 3). This implied that FA degrading activity is extant in *Variovorax* genera. *Variovorax* belongs to the recently identified *Comamonadaceae* family of bacteria [38] that thrive in fertile soils rich in organic matter mostly derived from plants [39, 40]. Thus, these bacteria should participate in degrading plant-derived materials in nature. *Variovorax* includes plant growth-promoting rhizobacteria that mutually interact with plants by producing enzymes that degrade plant hormone intermediates [41, 42]. The present study showed that *Variovorax* bacteria together with plants in the rhizosphere decomposed the plant-derived, physiologically active compound THFA that is oxidized to FA. The considerable accumulation of PA and DPA in cultured *Variovorax* raises the question of whether and how emerging groups of bacteria decompose these degradants in various environments.

## Conclusion

The mechanisms through which soil bacteria degrade synthetic folic acid (FA) have remained unexplored. The present study isolated the novel soil-bacterium *Variovorax* sp. F1, which produced carboxypeptidase G that liberated glutamate residues of FA and deaminofolic acids, and a pterin deaminase that deaminated FA and PA. We consider that both enzymes comprise the bacterial mechanism of FA degradation.

## Materials and methods

### Strains, culture, and media

Soil microorganisms that degrade FA were enriched by culture in Minimal M9 Medium comprising 10 mM KH_2_PO_4_, 10 mM KCl, 20 mM NH_4_Cl, 10 mM MgSO_4_, and 0.1% trace elements [43] (pH 7.2) (M9-FA medium) with 10 mM FA added as a carbon source. We replaced FA with PA, DPA and DFA (10 mM each in 0.1 M NaOH) in some cultures. Samples (0.1 g) from a weed rhizosphere (Table S1) were aerobically cultivated at 28 °C in 3 mL of M9-FA medium in 20-mL test tubes for 24 h with agitation at 120 rpm. Thereafter, cultures (30 μL) in 3 mL of fresh M9-FA were passaged at least four times under the same conditions, then broth from the enriched cultures and soil samples was spread over M9-FA agar plates. Isolates were cultured in Luria-Bertani (LB) medium overnight, then 1% of each was inoculated into 500-mL flasks containing 100 mL of M9-FA medium at 28 °C with agitation at 120 rpm. Bacterial growth was measured as total protein in culture pellets using the Protein Assay Dye Reagent (Bio-Rad Laboratories, Hercules, CA, USA) as described by the manufacturer. Briefly, total proteins were stained using the Bradford reagent, and concentrations were determined as Coomassie Blue dye absorption at 595 nm and compared with a standard curve of bovine serum albumin.

### Determination of FA, PA, DFA and DPA

Samples were dissolved in 0.1 M NaOH containing 1 M NaCl (pH 12.5), and centrifuged at 20,400×*g* and 4 °C for 5 min to remove insoluble materials before separation by anion exchange HPLC under the following conditions: column, TSKgel SAX column (6.0 mm × 15.0 cm) (Tosoh Bioscience, Tokyo, Japan); linear gradient of 100 to a 60:40 ratio of aqueous 0.1 M NaOH containing 1 M NaCl (pH 12.5) to 50% acetonitrile in 0.1 M NaOH; column temperature, 30 °C; flow rate, 1.0 mL min^− 1^. Folic acid, PA, DFA and DPA were detected in eluates as absorption at 254 nm using a 1260 Infinity system equipped with a photodiode array detector (Agilent Technologies, Santa Clara, CA, USA).

### Preparation of DFA and DPA

Folic acid (0.88 g) in 0.1-L 50 mM Tris-HCI (pH 7.5) was incubated with 5 μg mL^− 1^ *Variovorax* sp. F1 rPDA at 30 °C for 12 h. The rPDA was denatured with 1 M NaOH, then the pH of the reaction was reduced to < 4.0 with 1 M HCl to precipitate DFA with > 99% purity. Deaminopteroic acid was produced by incubating the DFA (~ 0.5 g) with 5 μg mL^− 1^ rCPG in 0.1-L 50 mM Tris-HCI (pH 7.5) containing 0.2 mM ZnSO_4_ at 30 °C for 6 h, then purified as described above to > 99%. The DFA and DPA were confirmed by liquid chromatography-mass spectrometry (LCMS). An LCMS 8030 spectrometer (Shimadzu Co., Kyoto, Japan) was equipped with a Purospher® STAR RP-18 endcapped column (particle size 5 μm, Merck KGaA, Darmstadt, Germany) and the flow rate of a 40-min linear gradient from 0 to 40% acetonitrile in 0.05% formic acid was 0.8 mL min^− 1^. Mass ions were detected in the negative mode under the following conditions: probe voltage, 3.5 kV; detection range, *m/z* = 10–500 for DFA (precursor *m*/*z* 441) and 10–400 (precursor *m*/*z* 312) for DPA; column temperature, 40 °C; desolvation line temperature, 250 °C; heat block temperature, 400 °C; nebulizer gas, 3 L min^− 1^; drying gas, 15 L min^− 1^.

### Protein sequence alignments and phylogenetic analysis

Amino acid sequences obtained from GenBank databases were aligned using CLUSTAL W [44]. A phylogenetic tree was constructed using MEGA X [34] and the neighbor-joining method [33] with 1000 bootstrap resampling replicates. Amino acid sequences with > 44% similarity to CPG in the F1 strain were selected from the Kyoto Encyclopedia of Genes and Genomes (KEGG) [45], and representative sequences were selected from redundant sequence pools derived from strains without species names. Amino acid sequences related to the F1 PDA were selected from *Variovorax paradoxus* S110 proteins in the KEGG database.

### Preparation of recombinant CPG (rCPG) from *Variovorax* sp. F1

Nucleotide sequences of putative CPG2 genes from various *Variovorax* bacteria were compared with conserved sequences among the genes using the Basic Local Alignment Search Tool (BLAST), and the (5’→3′) primers: ACCATCATCACCACAGCCAGGATCCGATGCGTCCGAGCATCCAT and TTAAGCATTATGCGGCCGCAAGCTTTCATTTGCCAGCAC. A DNA fragment encoding the CPG of the *Variovorax* sp. F1 CPG gene was amplified by PCR in a mixture containing these primers, bacterial total DNA, and Ex Taq® DNA Polymerase (Takara, Kyoto, Japan) at 94 °C for 5 min followed by 30 cycles of 98 °C for 10 s, 55 °C for 30 s, 72 °C for 1 min with an additional 7 min at 72 °C for the final cycle. The amplified DNA was fused to pRSFDuet-1 (Merck KGaA) and digested with *Bam*HI and *Hin*dIII using NEBuilder HiFi DNA Assembly Master Mix (New England Biolabs, Inc., Ipswich, MA, USA). *Escherichia coli* BL21 (DE3) (Merck KGaA), harboring the fused plasmid was incubated in LB medium for 12 h, then portions (1 mL) were cultured in 100 mL of fresh LB medium at 37 °C until the OD_600_ reached 0.5–0.6. Isopropyl β-d-1-thiogalactopyranoside (IPTG; final concentration, 0.1 mM) was added to induce rCPG production, then cultures were shaken for 18 h at 80 rpm and 28 °C.

Cells were harvested by centrifugation at 6,500×*g* for 10 min at 4 °C, washed twice with 5 mL of 20 mM sodium phosphate (pH 7.4) and 20 mM imidazole, then sonicated on ice for 200 s at 30% output on a 35% duty cycle using a Branson Sonifier® 250 (Branson Ultrasonics Corp., Brookfield, CT, USA). After centrifugation for 10 min at 6,500×*g*, the supernatant was applied to a 1-mL HisTrap™ FF crude column (GE Healthcare, Chicago, IL, USA), washed with 20 mM sodium phosphate (pH 7.4) containing 0.2 M NaCl and 20 mM imidazole, then rCPG was eluted with 20 mM sodium phosphate (pH 7.4) containing 0.2 M NaCl and 200 mM imidazole. The eluates were concentrated to 1 mL and the solvents were replaced with 20 mM Tris-HCl (pH 7.4) using an Amicon® Ultra-4 Centrifugal Filter Unit Ultracel-30 (Merck KGaA). Proteins were resolved by SDS-PAGE as described by Laemmli [46].

### Isolation of *Variovorax* sp. F1 PDA gene and preparation of rPDA

Orthologs to the predicted deaminase conserved among multi-*Variovorax* species (WP 068679287) were compared with the predicted genes of *V. paradoxus* strains and their nucleotide sequences. Conserved sequences were extracted to design the (5’→3′) primers ATGAAGCTCGAGGCCGTCCGC and TACTCGTACCCGTTCGGTTAC respectively corresponding to the 5′ ends of the *V. paradoxus* genes and the downstream endoribonucleotidase gene. We amplified a DNA fragment encoding the *Variovorax* sp. F1 PDA gene by PCR using the same primers, total DNA and other conditions used to amplify the CPG genes. The PDA genes were amplified using the rPDA primers ATTTCATATGAAGCTCGAGGCCGTCCGC and CTAACTCGAGTCATGCAATGT TCTCCTGTGA, then amplicons were digested with *Nde*I and *Xho*I, cloned into pET28a (Novagen, Madison, WI, USA), and introduced into *E. coli* BL21 (DE3). Transformants were cultured in LB medium at 30 °C until the OD_600_ reached 0.5–0.6, after which IPTG (final concentration, 0.2 mM) was added to induce gene expression overnight at 25 °C. The rPDA was purified as described for rCPG.

### Enzyme assays of rCPG and rPDA

Enzyme reactions of CPG proceeded in 50 mM Tris-HCl (pH 7.5) containing 0.2 mM ZnSO_4_ and appropriate amounts of rCPG at 30 °C, then the outcomes were analyzed by HPLC as described above. Substrates in the reaction buffer were incubated at 30 °C for 5 min, followed by reactions with a final concentration of 5 μg mL^− 1^ rCPG for 5 min. Enzyme reactions of rPDA proceeded in 50 mM Tris-HCl (pH 7.5) with appropriate amounts of rPDA at 30 °C, and were analyzed by HPLC under the same conditions. The enzyme concentration was determined by the Bradford method using Protein Assay Dye Reagent (Bio-Rad Laboratories, Hercules, CA, USA) as described by the manufacturer.

### Preparation and analysis of cell-free extract of *Variovorax* sp. F1

*Variovorax* sp. F1 was cultured in M9-FA medium at 28 °C for 24 h, harvested by centrifugation at 5100×*g* for 10 min, and washed with ice-cold 20 mM Tris-HCI (pH 7.2). Cells were resuspended in 5 mL of 50 mM Tris-HCI (pH 7.2) and disrupted as described above. The supernatants were filtered through a 0.45-μm CA syringe filter (Merck KGaA) to obtain cell-free extracts. The preparation (typically, 0.1 mg mL^− 1^ cell-free extract) in 50 mM Tris-HCl (pH 7.5) containing 0.2 mM ZnSO_4_ was reacted with purified FA, PA, DFA and AGB that were subsequently quantified by HPLC as described above.

## Supplementary Information

**Additional file 1: Fig. S1.** Determination of DFA and DPA. **Fig. S2.** Dependence of rCPG activity on substrate concentration.

**Additional file 2: Supplementary Table S1.** Bacteria isolated from grassland weed rhizosphere.

## Data Availability

The datasets generated during the current study are available in the GenBank repository (https://www.ncbi.nlm.nih.gov/genbank/, accession numbers MZ914412, LC718122, and MZ934696).

## Abbreviations

16S rRNA: 16S ribosomal RNA
ABG: *N*-(4-Aminobenzoyl)-L-glutamic acid
CPG: Carboxypeptidase G
DFA: Deaminofolic acid
DNA: Deoxyribonucleic acid
DPA: Deaminopteroic acid
FA: Folic acid
HPLC: High performance liquid chromatography
IPTG: Isopropyl β-D-1-thiogalactopyranoside
KEGG: Kyoto Encyclopedia of Genes and Genomes
LC-MS: Liquid chromatography-mass spectrometry
PA: Pteroic acid
PABA: *p*-aminobenzoic acid
PDA: Pterin deaminase
rCPG: Recombinant CPG
rPDA: Recombinant pterin deaminase
SDS-PAGE: Sodium dodecyl sulfate-polyacrylamide gel electrophoresis
THFA: Tetrahydrofolic acid
