# Supplementary material for: Carboxypeptidase G and pterin deaminase metabolic pathways degrade folic acid in Variovorax sp. F1
Source: BMC Microbiol. 2022 Sep 27;22:225. doi: 10.1186/s12866-022-02643-6 (PMC9513972; doi:10.1186/s12866-022-02643-6)
Supplement: Supplementary file 1 — Additional file 1: Fig. S1. Determination of DFA and DPA. Fig. S2. Dependence of rCPG activity on substrate concentration. [file 12866_2022_2643_MOESM1_ESM.docx]

**313**

**177**

**313**

**177**

A B

**DFA (*M*r = 442)**

**DPA (*M*r = 313)**

**441.15**

**312.15**

**136.00**

**176.05**

**269.10**

**312.15**

**Relative intensity**

**397.15**

**Relative intensity**

**176.00**

***m/z***

***m/z***

**Figure S1. Determination of DFA and DPA.**

Structures and MS-MS spectra of DFA and DPA obtained in negative ion mode.

**A**

**Specific activity**

**(μmol min^−1^ mg-protein^−1^)**

**B**

**C**

**Specific activity**

**(μmol min^−1^ mg-protein^−1^)**

**Specific activity**

**(μmol min^−1^ mg-protein^−1^)**

**FA (mM)**

**DFA (mM)**

**ABG (mM)**

**Figure S2. Dependence of rCPG activity on substrate concentration.**

A, FA; B, DFA; C, ABG.
