# Supplementary material for: Carboxypeptidase G and pterin deaminase metabolic pathways degrade folic acid in Variovorax sp. F1
Source: BMC Microbiol. 2022 Sep 27;22:225. doi: 10.1186/s12866-022-02643-6 (PMC9513972; doi:10.1186/s12866-022-02643-6)
Supplement: Supplementary file 2 — Additional file 2: Supplementary Table S1. Bacteria isolated from grassland weed rhizosphere. [file 12866_2022_2643_MOESM2_ESM.docx]

Supplementary Table S1. Bacteria isolated from grassland weed rhizosphere.

| Strain | Genus | Closest species | Homology* | Sampling location | |
| --- | --- | --- | --- | --- | --- |
|  |  |  |  | Physical | Cartographic |
| F1 | *Variovorax* | *V. paradoxus*  NBRC 15149 | 99.9% | Grassland weed rhizosphere | 36°06'30.3"N,  140°06'21.4"E |
| F2 | *Xenophilus* | *X. aerolatus*  5516S-2 | 99.4% | Grassland weed rhizosphere | 36°06'30.2"N,  140°06'19.2"E |
| F3 | *Variovorax* | *V. paradoxus*  NBRC 15149 | 99.5% | Grassland weed rhizosphere | 36°06'31.8"N,  140°06'22.4"E |
| F4 | *Xenophilus* | *X. aerolatus*  5516S-2 | 99.7% | Grassland weed rhizosphere | 36°06'26.3"N,  140°06'10.6"E |
| F5 | *Variovorax* | *V. paradoxus*  NBRC 15149 | 99.5% | Grassland weed rhizosphere | 36°06'41.6"N,  140°06'33.7"E |
| F6 | *Variovorax* | *V. paradoxus*  NBRC 15149 | 99.2% | Grassland weed rhizosphere | 36°06'47.5"N,  140°06'14.4"E |
| F7 | *Variovorax* | *V. paradoxus*  NBRC 15149 | 99.3% | Grassland weed rhizosphere | 36°06'37.3"N,  140°06'12.2"E |
| F8 | *Variovorax* | *V. paradoxus*  NBRC 15149 | 99.7% | Grassland weed rhizosphere | 36°06'42.7"N,  140°06'26.1"E |

*Sequence homology of 16S rRNA gene against closest species.
